# Genome-Wide and GWAS Dissection of Maize Fibrillin Genes Reveals Plastid Regulators of Drought and Salt Stress Tolerance

**DOI:** 10.3390/cimb48080819

**Published:** 2026-08-12

**Authors:** Suwen Han, Renjie Zhao, Jingpei Piao, Xingzheng Zhang, Miaomiao Liu, Liangxuan Jia, Jianfeng Liu, Yuejia Yin, Hanchao Xia

**Affiliations:** 1 Jilin Provincial Key Laboratory of Plant Resource Science and Green Production, Jilin Normal University, Siping 136000, China; hansuwen@webmail.hzau.edu.cn (S.H.); parkfreedom@126.com (J.P.); xz-zhang@jlnu.edu.cn (X.Z.); 15584118470@163.com (M.L.); 19543455132@163.com (L.J.); jianfengliu1976@163.com (J.L.); 2 National Key Laboratory of Crop Genetic Improvement, Huazhong Agricultural University, Wuhan 430070, China; 3 Institute of Crop Germplasm Resources, Jilin Academy of Agricultural Sciences (Northeast Agricultural Research Center of China), Gongzhuling 136100, China; renjie_z@126.com; 4 Institute of Agricultural Quality Standard and Testing Technology, Jilin Academy of Agricultural Sciences (Northeast Agricultural Research Center of China), Changchun 130033, China

**Keywords:** *ZmFBN* gene family, abiotic stress, GWAS, *Zea mays* L.

## Abstract

Fibrillins (FBNs) are conserved plastid-associated proteins implicated in plant development and abiotic stress responses; however, their roles in maize remain unclear. In this study, through a genome-wide bioinformatic analysis, we identified 14 *ZmFBN* genes in the maize genome and characterized their phylogeny, chromosomal distribution, gene structure, conserved motifs, and promoter cis-elements. *ZmFBN* members were grouped into several subfamilies that all retain a conserved PAP_fibrillin domain, whereas the variation in exon–intron organization, motif composition, and regulatory elements suggests functional diversification. Expression profiling revealed pronounced tissue-preferential patterns, with many genes highly expressed in leaves and reproductive tissues, and distinct responses to drought, salt, heat, and cold stresses. qRT-PCR assays showed that *ZmFBN8* and *ZmFBN9* are strongly induced by both salt and PEG-simulated drought, *ZmFBN2* and *ZmFBN5* are predominantly drought-responsive, and *ZmFBN11* is mainly activated by salt. Genome-wide association analysis further detected significant loci near *ZmFBN1* and *ZmFBN4*, whose allelic variants are associated with the survival rate under drought and with key agronomic traits, including the tassel branch number, flowering time, ear diameter, and kernel length. These results demonstrate that *ZmFBN* genes make diversified contributions to maize growth, development, and stress adaptation and highlight several members as promising targets for functional studies and the molecular breeding of stress-tolerant maize. Moreover, selection pressure analysis indicated *ZmFBN7* experienced relaxed purifying selection, and *ZmFBN12* underwent positive selection, which drives the functional diversification of the *ZmFBN* family during maize evolution.

## 1. Introduction

Maize (*Zea mays* L.) is one of the most important cereal crops worldwide and plays essential roles in food security, animal feed, and industrial production [1]. However, drought and salinity are major abiotic stresses that severely restrict maize growth, development, and yield formation [2,3]. Drought stress can markedly impair photosynthesis, osmotic adjustment, and biomass accumulation, while triggering extensive transcriptional reprogramming in maize tissues [4,5]. Salt stress further disrupts ion homeostasis, redox balance, and cellular metabolism, thereby reducing plant performance under unfavorable environments [6,7]. Therefore, identifying the key genes involved in both drought and salt stress responses and elucidating their regulatory mechanisms are important steps toward improving stress resilience in maize. Fibrillins (FBNs), also known as plastid lipid-associated proteins, are a conserved family of proteins widely distributed in photosynthetic organisms [8,9]. FBN proteins are mainly localized in plastoglobules and related plastid structures, where they contribute to plastid stability, carotenoid accumulation, lipid metabolism, and protection of the photosynthetic apparatus [10]. Recent studies have shown that the FBN family is not only involved in plant growth and development [11,12] but also participates broadly in responses to abiotic stresses, including drought, heat, cold, oxidative stress, and high light [13,14,15]. Because both drought and salinity directly affect chloroplast function, photosynthetic efficiency, and oxidative homeostasis, FBN genes are promising candidates for investigating the molecular basis of abiotic stress adaptation in maize.

Genome-wide studies of FBN gene families have been conducted in several plant species and have provided important insights into their evolutionary conservation and functional divergence [16,17,18,19,20,21]. In tomato, for example, FBN family members have been shown to share conserved PAP domains but differ in gene structure, conserved motifs, cis-acting elements, and expression profiles across tissues and treatments, suggesting functional diversification among family members [16]. Similar observations have also been reported in wheat, where many *TaFBN* genes respond to drought and other stresses, further supporting the involvement of this family in stress adaptation [21]. Despite these advances, systematic studies of the FBN gene family in maize remain limited, and the genomic organization, evolutionary characteristics, and stress-responsive expression patterns of *ZmFBN* genes are still poorly understood. In parallel with gene family studies, genome-wide association study (GWAS) has become a powerful approach for dissecting the genetic basis of complex stress resistance traits in maize [22,23,24]. Previous GWAS analyses have identified significant loci and candidate genes associated with seedling-stage drought tolerance, providing valuable clues regarding the genetic architecture underlying the adaptation of maize to water deficit [25]. Integrative strategies combining GWAS with transcriptomic analysis have further improved candidate gene prioritization and helped to reveal key drought-responsive regulators, such as *ZmGRAS15* [26]. Nevertheless, the contribution of the natural variation in FBN genes to drought-related traits in maize has not yet been systematically evaluated.

In this study, a genome-wide identification and characterization of the FBN gene family was performed in maize. The physicochemical properties, chromosomal distribution, phylogenetic relationships, gene structures, and conserved motifs of *ZmFBN* genes were systematically analyzed to clarify their evolutionary and structural features. In addition, promoter cis-acting elements and expression patterns in different tissues were examined to infer their potential biological functions. Considering that drought and salinity are two major environmental constraints in maize production, the expression profiles of *ZmFBN* genes under drought and salt treatments were further analyzed, and representative candidate genes were validated using qPCR. Moreover, drought-related GWAS results were integrated with expression evidence to screen potential *ZmFBN* candidate genes associated with drought tolerance. These findings provide a foundation for further functional analysis of *ZmFBN* genes and offer candidate targets for improving abiotic stress resistance through maize breeding.

## 2. Materials and Methods

### 2.1. Identification of the ZmFBN Gene Family and Phylogenetic Analysis Across Plant Species

To systematically identify members of the FBN gene family in maize, the hidden Markov model (HMM) profile of the PAP_fibrillin domain (PF04755) was downloaded from the Pfam database [27] and was used as a query to search the complete protein datasets of *Zea mays* B73 RefGen_v5, *Setaria italica*, *Sorghum bicolor*, and *Arabidopsis thaliana* using the hmmscan program in the HMMER software package (version 3.3.2) [28]. Maize genome and protein sequence data were obtained from the Ensembl Plants database [29], while *Arabidopsis thaliana* protein sequences were downloaded from TAIR10. Protein datasets of the other species were also retrieved from Ensembl Plants. The e-value threshold in hmmscan was set to 1 × 10^−15^ to retain only proteins with significant matches to the FBN domain.

The candidate protein sequences were further examined using Pfam (https://bio.tools/pfam, accessed on 4 August 2026) and InterProScan (https://www.ebi.ac.uk/interpro/search/, accessed on 4 August 2026) to confirm the presence of complete FBN-related conserved domains and to remove redundant or incorrectly annotated sequences. After filtering, a high-confidence set of FBN family members was obtained. Multiple sequence alignment of FBN proteins from the four species, together with an FBN homolog (NCBI accession XP_042915411.1; gene locus CHLRE_16g649200v5, *Chlamydomonas reinhardtii* v5.6) from *Chlamydomonas reinhardtii* as the outgroup, was then performed using the MUSCLE algorithm in MEGA [30] under the default parameters. The best-fit amino acid substitution model was selected based on the minimum Bayesian Information Criterion (BIC) using the model selection function in MEGA12. The optimal model JTT+G+I+F was adopted to construct the maximum-likelihood (ML) phylogenetic tree. Gamma-distributed rate variation among sites (+G), a proportion of invariant sites (+I), and empirical amino acid frequencies (+F) were incorporated. Pairwise deletion was employed to handle gaps and missing data during tree reconstruction. Branch support was evaluated with 1000 standard bootstrap replicates. The final phylogenetic tree was visualized and annotated using TBtools (version 2.475) [31].

### 2.2. Chromosomal Localization and Collinearity Analysis of ZmFBN Genes

The maize reference genome sequence and corresponding GFF3 annotation file were downloaded from the Ensembl Plants database. The chromosomal positions of *ZmFBN* genes were extracted from the GFF3 annotation and visualized using TBtools. To investigate the duplication pattern and collinearity relationships of the *ZmFBN* family within the maize genome, whole-genome self-comparison was conducted using MCScanX integrated in TBtools. Syntenic blocks and duplicated regions were identified based on genomic collinearity analysis. The collinearity relationships among *ZmFBN* genes and across the maize genome were then displayed using Circos (version 0.69.8) [32].

### 2.3. Calculation of Ka and Ks Values Among NAM Orthologous Sequences

The protein and coding sequences (CDSs) of *ZmFBN* genes in the maize NAM founder lines were obtained from public maize NAM genomic resources, as described previously [33]. The B73 reference sequences were used as queries to identify one-to-one *ZmFBN* orthologs in each NAM founder line, and the corresponding CDS pairs were aligned in a codon-aware manner based on their protein sequence alignments. Pairwise nonsynonymous (Ka) and synonymous (Ks) substitution rates, as well as Ka/Ks ratios, were then calculated using the KaKs_Calculator (version 3.0) [34] with the default settings.

### 2.4. Gene Structure, Conserved Motifs, Protein Domains, and cis-Acting Element Analysis of ZmFBN Genes and Proteins

The exon–intron structures of *ZmFBN* genes were extracted from the GFF3 annotation file and visualized using TBtools. Conserved motif analysis was performed on the *ZmFBN* protein sequences using the MEME Suite (https://meme-suite.org/meme/, accessed on 4 August 2026) [35]. The maximum number of motifs was set to 15, and the motif width ranged from 6 to 50 amino acids. The distribution and composition of motifs among different *ZmFBN* proteins were then compared.

Protein domain analysis was carried out using InterProScan [36] to identify conserved domains within *ZmFBN* proteins. The detected domains were further compared with Pfam annotations to confirm the integrity of FBN family members.

For promoter analysis, the 1000 bp genomic sequence upstream of the translation start site of each *ZmFBN* gene was extracted as the putative promoter region. These sequences were submitted to the PlantCARE database [37] to identify cis-acting regulatory elements. Particular attention was given to elements associated with light responsiveness, hormone responsiveness (including abscisic acid, methyl jasmonate, and salicylic acid), defense and stress responses, low-temperature responsiveness, and seed- or endosperm-specific expression. Gene structure, motif composition, domain architecture, and cis-element distribution were all visualized using TBtools.

### 2.5. Expression Analysis of ZmFBN Genes in Different Tissues and Under Abiotic Stress Conditions

To examine the expression characteristics of *ZmFBN* genes during maize growth, development, and abiotic stress responses, transcriptome data from different tissues and developmental stages were retrieved from two independent sources. Tissue expression data (covering 15 tissue/developmental stage categories) and multi-stress expression data (cold, drought, heat, and salt stress treatments) were obtained from the qTeller database of MaizeGDB [38]. Drought-gradient RNA-seq data (well-watered, WW; and three progressive drought treatments, DT2, DT3, DT4) were retrieved from a previous study [39] (NCBI BioProject: PRJNA511671; SRA: SRP174413; GEO: GSE124340). In brief, wild-type maize B73 seeds were sown four per pot and grown under well-watered (WW) conditions (65–75% soil moisture) for approximately 15 days. Progressive drought was then imposed by withholding water until the designated soil moisture levels were reached, after which soil moisture was maintained by daily watering. Four conditions were included: WW (well-watered, 65–75%), DT2 (mild drought, 30–35%), DT3 (moderate drought, 20–25%), and DT4 (severe drought, 10–15%). Maize seedlings were harvested on treatment day 7, when they had approximately four true leaves. The absolute FPKM values of all *ZmFBN* genes across all samples are provided in Supplementary Table S1.

All expression data were log_2_-transformed and row-normalized before analysis. Heatmaps were generated using TBtools to visualize the expression patterns of *ZmFBN* genes across tissues, drought-gradient treatments, and multiple abiotic stress conditions. Hierarchical clustering analysis was also performed to evaluate similarities in expression patterns among *ZmFBN* genes.

### 2.6. qRT-PCR Validation of ZmFBN Genes Expression Under Salt and Drought Stress

To validate the transcriptome-based expression patterns, *ZmFBN* genes were analyzed using qRT-PCR under salt and PEG-simulated drought stress treatments. Maize seedlings were grown under strictly temperature-controlled greenhouse conditions at 25 °C with a 16/8 h light/dark photoperiod. To induce salt stress, three-leaf-stage plants were irrigated with 200 mM NaCl solution. For drought stress, three-leaf-stage seedlings were subjected to drought stress simulated by applying 20% PEG 6000 (Sinopharm Chemical Reagent Co., Ltd., Shanghai, China). Leaf samples were collected from the upper third of the third leaf of three individual seedlings at 0, 4, 6, and 12 h post-treatment, immediately snap-frozen in liquid nitrogen, and subsequently stored at −80 °C for subsequent RNA extraction. Three biological replicates were collected per time point per treatment. Total RNA was extracted from maize tissues using the FastPure Universal Plant Total RNA Isolation Kit (Vazyme, Nanjing, China). RNA integrity was assessed by 1.5% agarose gel electrophoresis, and RNA concentration and purity (A260/A280 and A260/A230 ratios) were determined using a NanoDrop ND-2000 spectrophotometer (Thermo Fisher Scientific, Wilmington, DE, USA). Complementary DNA (cDNA) was synthesized using TransScript^®^ One-Step gDNA Removal and cDNA Synthesis SuperMix (TransGen Biotech, Beijing, China).

To investigate the expression patterns of *ZmFBN* genes under drought and salt stress conditions, quantitative real-time PCR (qRT-PCR) analysis was performed. Gene-specific primers were designed using the NCBI Primer-BLAST tool (https://www.ncbi.nlm.nih.gov/tools/primer-blast/index.cgi?/, accessed on 1 October 2025) with the default maize reference genome as the template. qRT-PCR was carried out using ChamQ SYBR qPCR Master Mix (Vazyme Biotech Co., Ltd., Nanjing, China) on a StepOnePlus^TM^ Real-Time PCR System (Thermo Fisher Scientific, Foster City, CA, USA). Each cDNA sample was run in three technical replicates. Relative gene expression was calculated using the relative quantification (RQ) method, and statistical significance was determined by one-way ANOVA followed by Tukey’s honestly significant difference (HSD) post hoc test for multiple comparisons, with *p* < 0.05 considered statistically significant. Details of the primers employed are provided in Table S2.

### 2.7. GWAS-Based Association Analysis of Candidate ZmFBN Genes

To evaluate the potential contribution of *ZmFBN* genes to drought-related traits in a natural maize population, genome-wide association analysis was performed using genotype and phenotype data from a maize association panel [40,41]. Maize agronomic traits and drought tolerance traits were also collected from previous studies [42].

GWAS was conducted in Tassel5 [43] using a mixed linear model (MLM), with both population structure and kinship incorporated to reduce the effects of population stratification and relatedness. The significance threshold was set to *p* < 1/*n* (total number of SNPs tested) based on Bonferroni correction for multiple testing across the genome. Significant SNPs located within 5 kb upstream or downstream of *ZmFBN* genes were regarded as candidate association loci.

For key candidate genes, such as *ZmFBN1* and *ZmFBN4*, local linkage disequilibrium analysis was further performed using SNPs surrounding the target region. Pairwise *r*^2^ values were calculated, and local LD heatmaps were drawn in R to examine the haplotype structure around the candidate loci. In addition, the natural population was grouped according to representative SNP genotypes, and differences in the survival rate, gene expression, and agronomic traits among allelic groups were compared to evaluate the effects of different alleles on drought tolerance and yield-related traits. The GWAS results and LD structures were visualized in R (version 4.6.10).

### 2.8. Upstream Transcription Factors’ Prediction of the ZmFBNs

ChIP-seq data from prior research [44] (NCBI BioProject: PRJNA518749; GEO: GSE137972) involving 104 transcription factors (TFs) were employed to predict the upstream transcription factors of the *ZmFBN* family genes, and the interactions between these transcription factors and their target genes were visualized using the Gephi (https://gephi.org/) software (version 0.9.7).

## 3. Results

### 3.1. Identification, Phylogenetic Classification, and Chromosomal Distribution of ZmFBNs

A total of 14 FBN family genes were identified in the maize genome, named *ZmFBN1*–*ZmFBN14* (Supplementary Table S3). To clarify their evolutionary relationships, a phylogenetic tree was constructed using FBN protein sequences from maize, Arabidopsis, sorghum, and *Setaria italica*. The resulting tree showed that FBN proteins from different species were distributed across several distinct clades, and the maize members were grouped together with homologs from both monocot and dicot species, rather than forming a completely independent lineage, indicating that the FBN family is evolutionarily conserved in higher plants, and its major diversification likely occurred before the divergence of these species.

**Figure 1:**
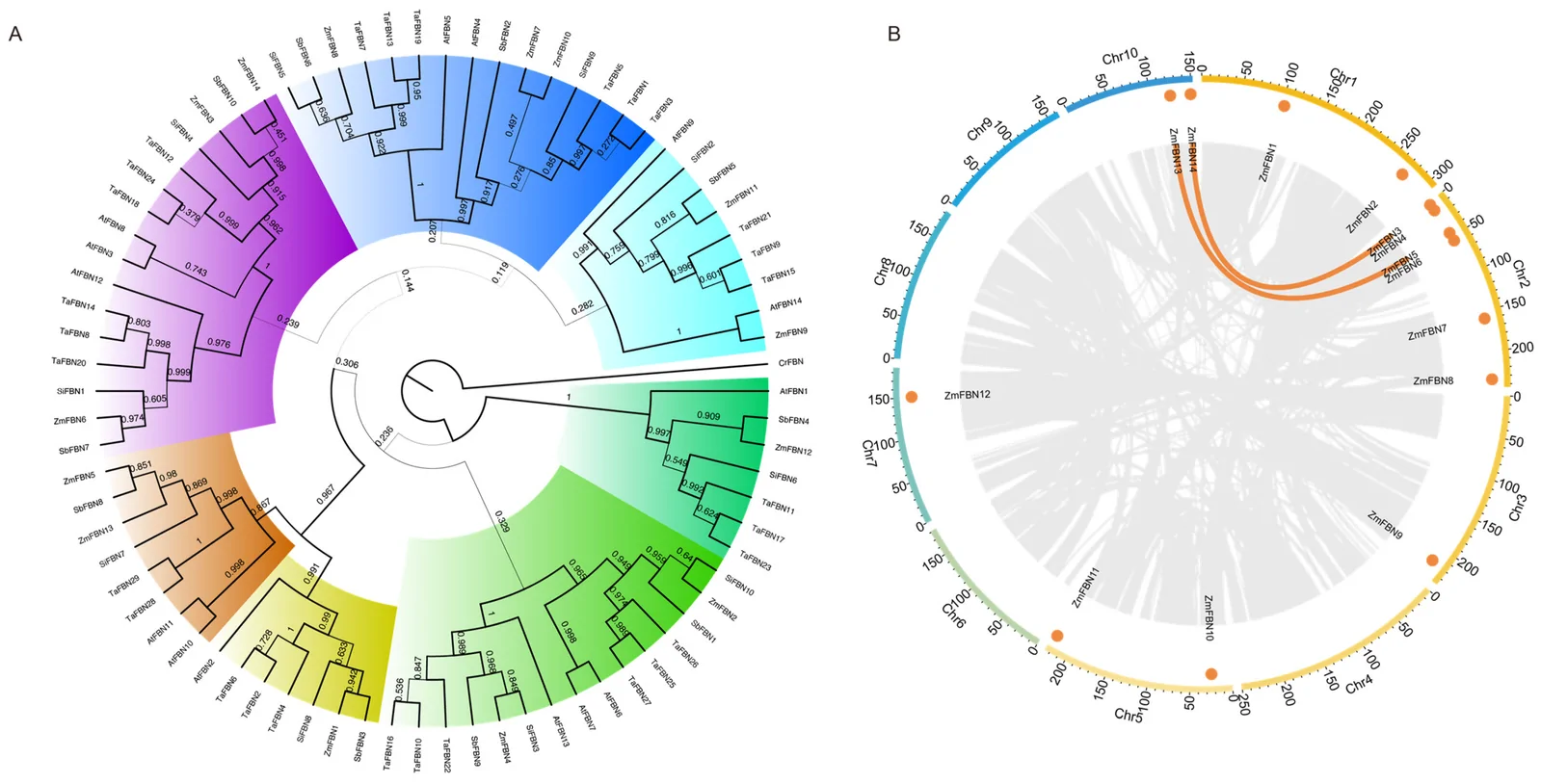
Phylogenetic analysis of fibrillin (FBN) proteins from different species and the chromosomal localization of maize *ZmFBNs.* (**A**) Phylogenetic tree of FBN proteins from *Zea mays* (Zm), *Arabidopsis thaliana* (At), *Sorghum bicolor* (Sb), and *Setaria italica* (Si) with a *Chlamydomonas reinhardtii* homolog as the outgroup. Clade backgrounds are colored to distinguish different FBN subfamilies; Bootstrap values (1000 replicates) are labeled on the main branches. (**B**) Chromosomal localization map of *ZmFBN* genes. Chromosome numbers are labeled next to each chromosome segment; the ten maize chromosomes are colored for visual distinction, orange dots mark the physical positions of *ZmFBN* genes, orange lines represent the collinear relationships between *ZmFBN* gene pairs, and gray lines represent both intrachromosomal and interchromosomal collinear relationships of the entire maize genome.

Within the phylogenetic tree (Figure 1A), the 14 *ZmFBN* proteins were distributed among multiple subgroups, suggesting that the maize FBN family has undergone a certain degree of functional differentiation during evolution. Some *ZmFBN* members clustered closely with sorghum and *Setaria italica* homologs, implying that these genes may retain conserved functions in monocot species, whereas others showed relatively distinct branching patterns, indicating possible lineage-specific divergence.

Chromosomal localization analysis further showed that the 14 *ZmFBN* genes were unevenly distributed across several maize chromosomes rather than concentrated in a single genomic region (Figure 1B). Synteny analysis revealed clear collinear relationships among some family members, particularly between *ZmFBN1*, *ZmFBN6*, and *ZmFBN14*, suggesting that segmental duplication contributed to the retention and expansion of part of the family. However, no large-scale tandem duplication clusters were observed, indicating that the maize FBN family is relatively conserved in copy number and has experienced limited expansion overall. Taken together, these results suggest that the *ZmFBN* family evolved in a generally conserved manner, while a subset of genes may have diverged after duplication events.

**Figure 2:**
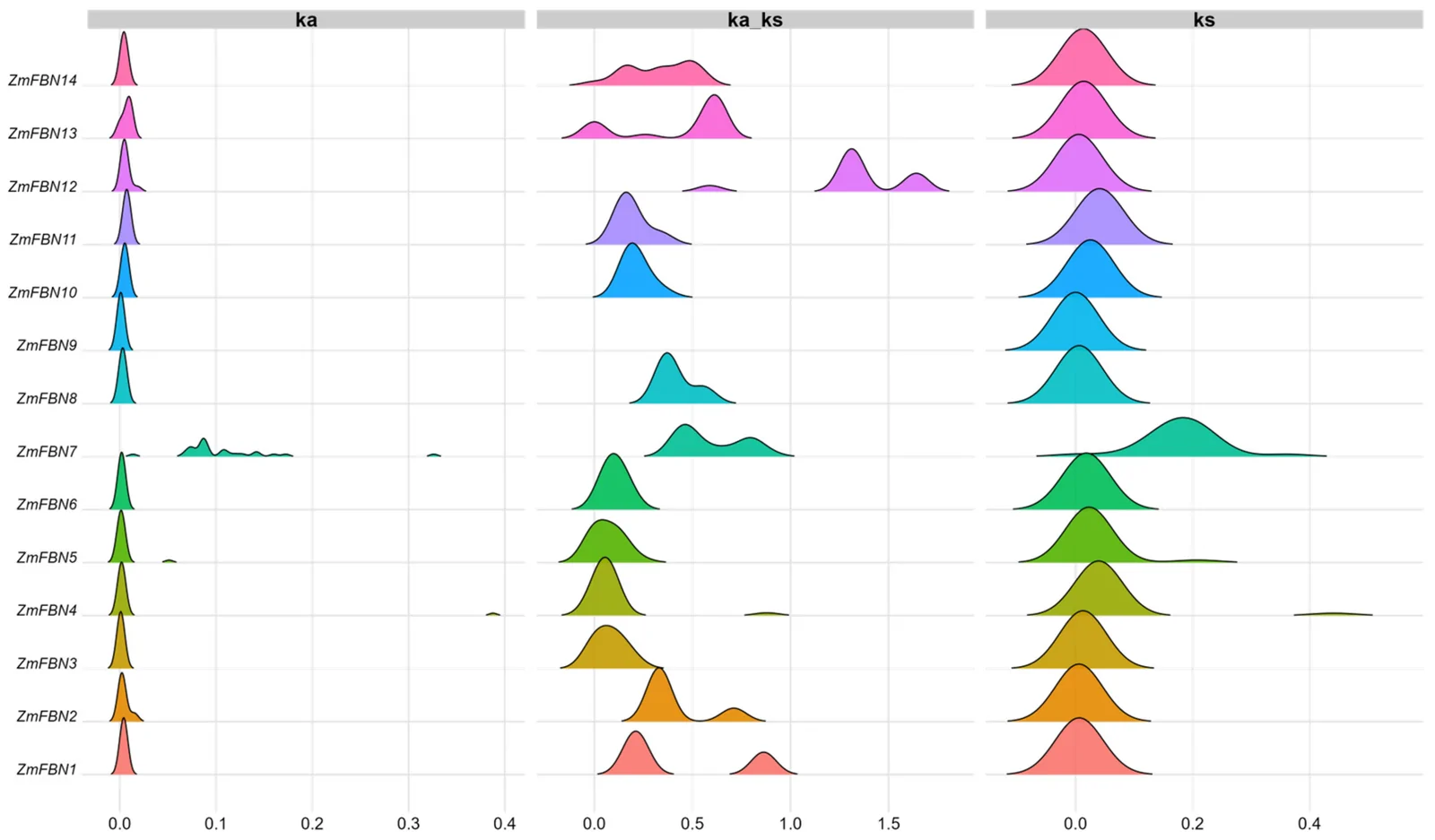
Distributions of Ka, Ka/Ks, and Ks for *ZmFBN* homologs in NAM founder lines. Each ridge represents the distribution of the pairwise nonsynonymous substitution rate (Ka), synonymous substitution rate (Ks), or their ratio (Ka/Ks) for one *ZmFBN* gene between the B73 reference and its orthologs in maize NAM founder lines. Ridges are colored according to the individual *ZmFBN* genes, as indicated on the y-axis.

To clarify the evolutionary constraints acting on the *ZmFBN* gene family, we calculated nonsynonymous (Ka) and synonymous (Ks) substitution rates and the corresponding Ka/Ks ratios between B73 and orthologs from NAM founder lines and visualized the parameter distribution via ridgeline plots (Figure 2). Most *ZmFBN* members displayed compact Ka and Ks density peaks close to the zero axis with overall Ka/Ks far below 1, indicative of dominant strong purifying selection and highly conserved coding sequences across the maize germplasm.

Two distinct evolutionarily differentiated genes were observed from the distribution profiles: *ZmFBN7* possessed markedly broadened and right-shifted Ka/Ks distribution, representing the most obvious relaxation of purifying selection across the entire gene, with all values remaining below 1. *ZmFBN12* was the sole member carrying partial Ka/Ks values above 1, a typical signature of positive selection during maize intraspecific evolution.

Taken together, the *ZmFBN* family is predominantly shaped by purifying selection to conserve core plastid-associated biological functions, whereas the relaxed selection on *ZmFBN7* and positive selection on *ZmFBN12* drive the sequence variation and functional diversification among paralogs.

### 3.2. Gene Structure, Conserved Motifs, Domains, and cis-Acting Elements of ZmFBNs

To further characterize the structural features of the *ZmFBN* family, the gene structure, conserved motifs, protein domains, and promoter cis-acting elements were analyzed in combination with phylogenetic relationships. Overall, the gene structures of *ZmFBN* members were not identical, but genes clustered in the same phylogenetic branch generally exhibited similar exon–intron organization. Some members displayed relatively compact structures with fewer exons, whereas others contained more complex exon arrangements and longer genomic regions, indicating structural divergence within the family. This structural variation suggests that different *ZmFBN* genes may differ in transcriptional regulation complexity or functional specialization.

**Figure 3:**
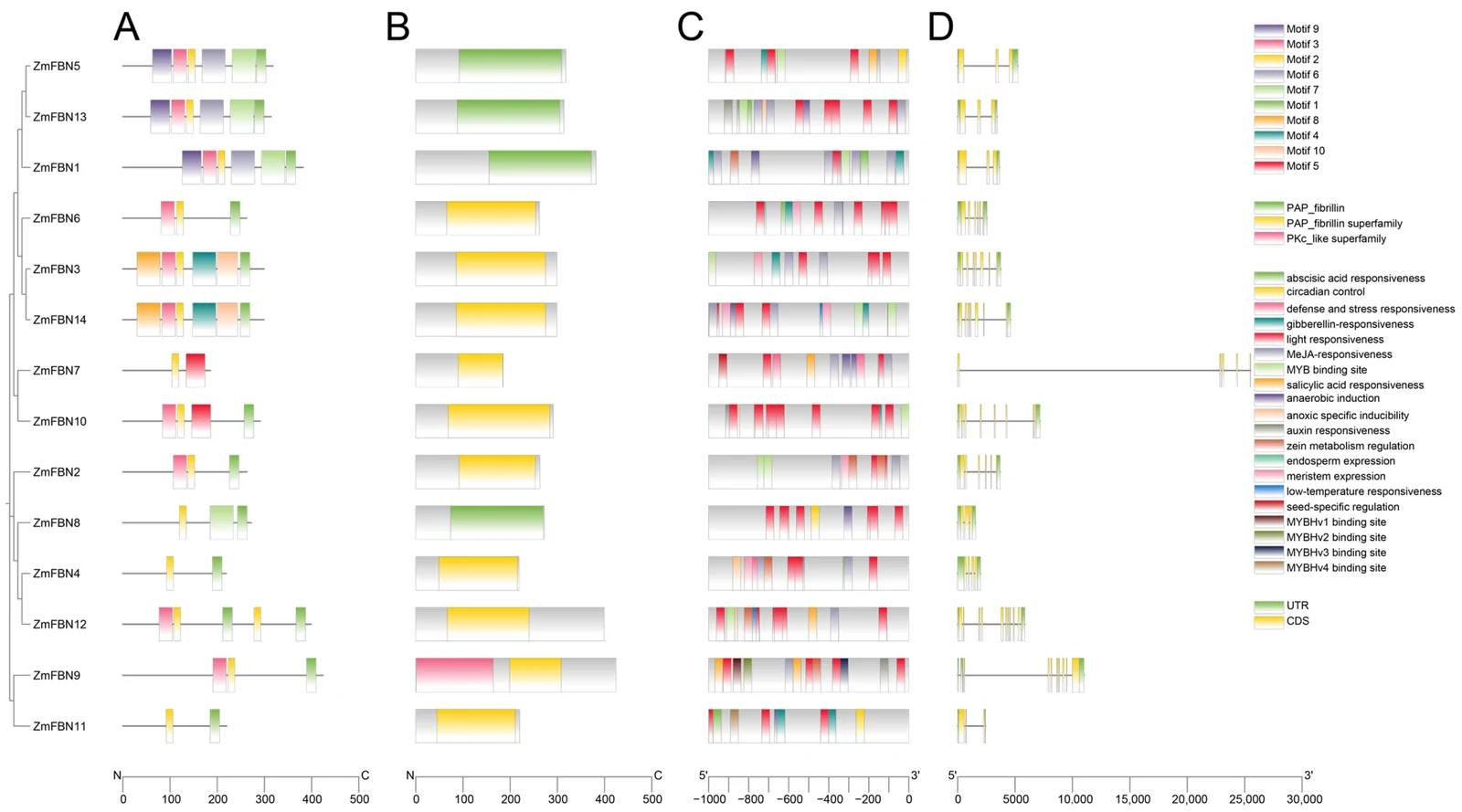
Phylogeny, conserved motifs, protein domains, cis-acting regulatory elements, and gene structure of *ZmFBN* family genes from left to right. (**A**) Conserved motif analysis, where different colors represent different motifs, showing the distribution pattern of motifs among family members; (**B**) protein domain analysis, where different colors label the conserved domains contained in each member; (**C**) analysis of cis-acting regulatory elements in promoters, labeling regulatory elements related to hormone response, stress response, and light response in the 1000 bp upstream region, (*x*-axis: −1000 to 0 bp, with the transcription start site at position 0); (**D**) gene structure analysis, where yellow rectangles represent exons (CDS), green rectangles represent untranslated regions (UTRs), and black lines represent introns.

Motif analysis showed that the *ZmFBN* proteins shared several highly conserved motifs (Figure 3A), supporting the conserved nature of the family. Meanwhile, differences in the number, distribution, and arrangement of motifs were also observed among members, especially between genes belonging to different phylogenetic branches. Members within the same subgroup tended to possess similar motif combinations, implying that they may have related protein structures and similar biological functions, whereas a few genes showed motif loss or simplified motif composition, suggesting possible functional divergence.

Protein domain analysis further demonstrated that most *ZmFBN* proteins contained the typical PAP_fibrillin or PAP_fibrillin superfamily domain (Figure 3B), consistent with their identity as plastid lipid-associated proteins. A few members also carried other domain features, implying that some *ZmFBN* proteins may participate in more specialized biological processes or protein interaction networks.

Promoter analysis revealed that the upstream regions of *ZmFBN* genes harbor abundant cis-acting elements associated with light responsiveness (Figure 3C), abscisic acid responsiveness, MeJA responsiveness, salicylic acid responsiveness, defense and stress responses, low-temperature responsiveness, and developmental regulation. MYB-related binding sites and elements associated with anaerobic induction, endosperm expression, seed-specific regulation, and zein metabolism regulation were also detected in several genes. Gene structure analysis (Figure 3D) further revealed high conservation in coding sequences (CDS, yellow rectangles) across members, ensuring the stability of core functional domain coding information, while significant variation was observed in untranslated regions (UTRs, green rectangles) and exon–intron architectures.

These findings indicate that *ZmFBN* genes may be involved not only in plastid-related basal physiological functions but also in hormone-mediated regulation, stress adaptation, and tissue-specific developmental processes. Overall, the *ZmFBN* family displays a pattern of conserved core structure together with diversified regulatory potential.

### 3.3. Expression Profiles of ZmFBN Genes in Different Tissues and Under Abiotic Stresses

To explore the potential functions of *ZmFBN* genes in maize growth, development, and stress adaptation, their expression patterns were analyzed across multiple tissues, under drought-gradient treatments and under different abiotic stresses, followed by qPCR validation of selected candidates. In general, the *ZmFBN* family exhibited clear spatiotemporal expression divergence, suggesting that its members play distinct roles in both developmental and stress-responsive processes.

**Figure 4:**
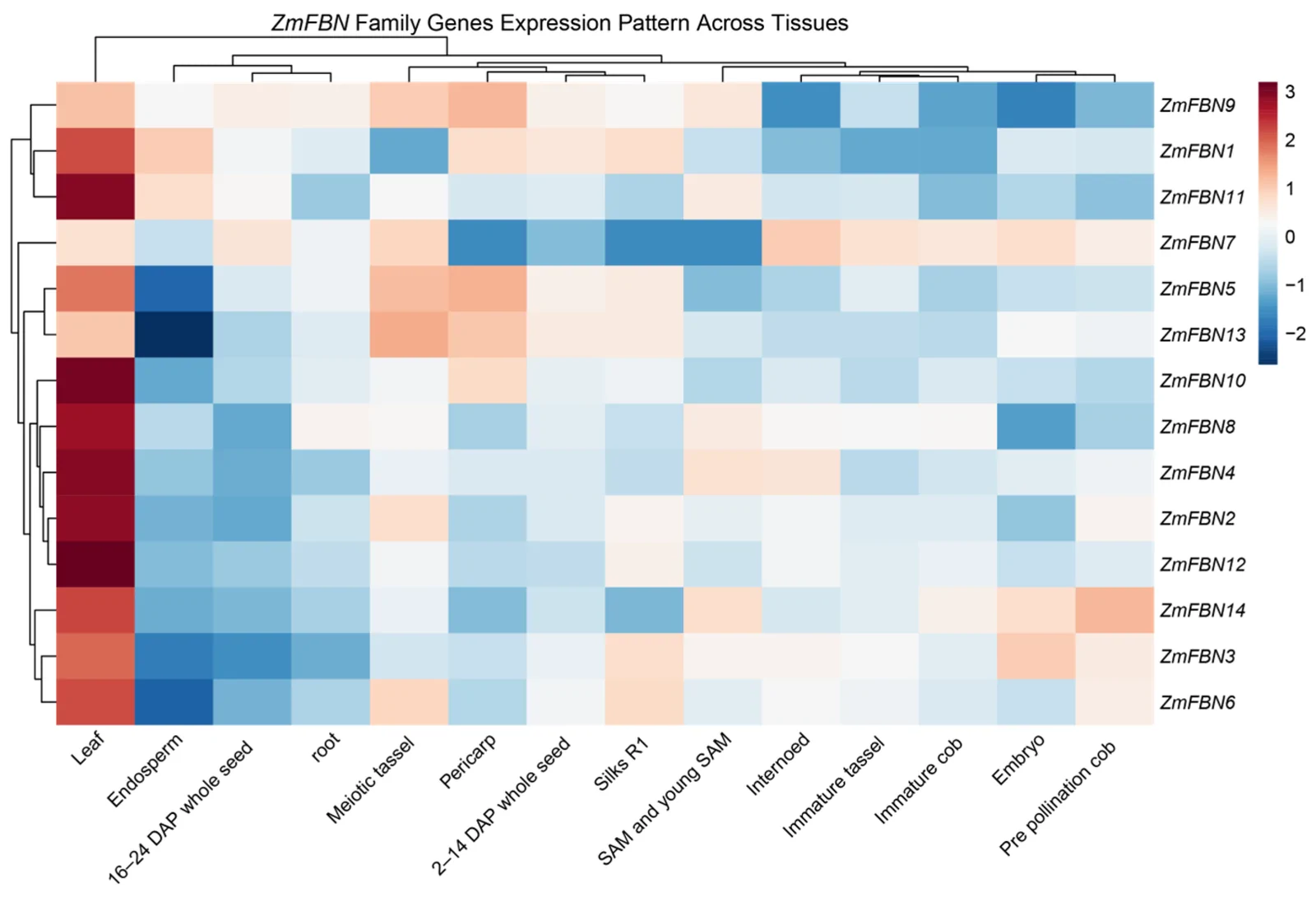
Expression profiles of *ZmFBN* genes across different tissues and developmental stages of maize. Expression data were obtained from the MaizeGDB qTeller database. The color scale indicates the expression level (log_2_(FPKM + 1)), with red and blue representing high and low expression, respectively.

Tissue expression analysis revealed that most *ZmFBN* genes exhibited obvious tissue-preferential expression rather than a uniform transcription pattern (Figure 4). Several members were highly expressed in leaves, suggesting potential roles in chloroplast development, maintenance of photosynthetic function, and photoprotection. In addition to leaves, some genes also showed relatively high expression in meiotic tassel, pericarp, silks_R1 (where R1 refers to the silking stage when silks first emerge from the husk), SAM and young SAM, immature tassel, embryo, and pre-pollination cob, implying that *ZmFBN* genes may participate not only in plastid-related functions in vegetative tissues but also in reproductive development, tassel differentiation, and kernel-related developmental processes. Together with the presence of seed- and endosperm-related cis-elements in the promoters of several members, these expression patterns further support the functional divergence among *ZmFBN* genes during maize development.

**Figure 5:**
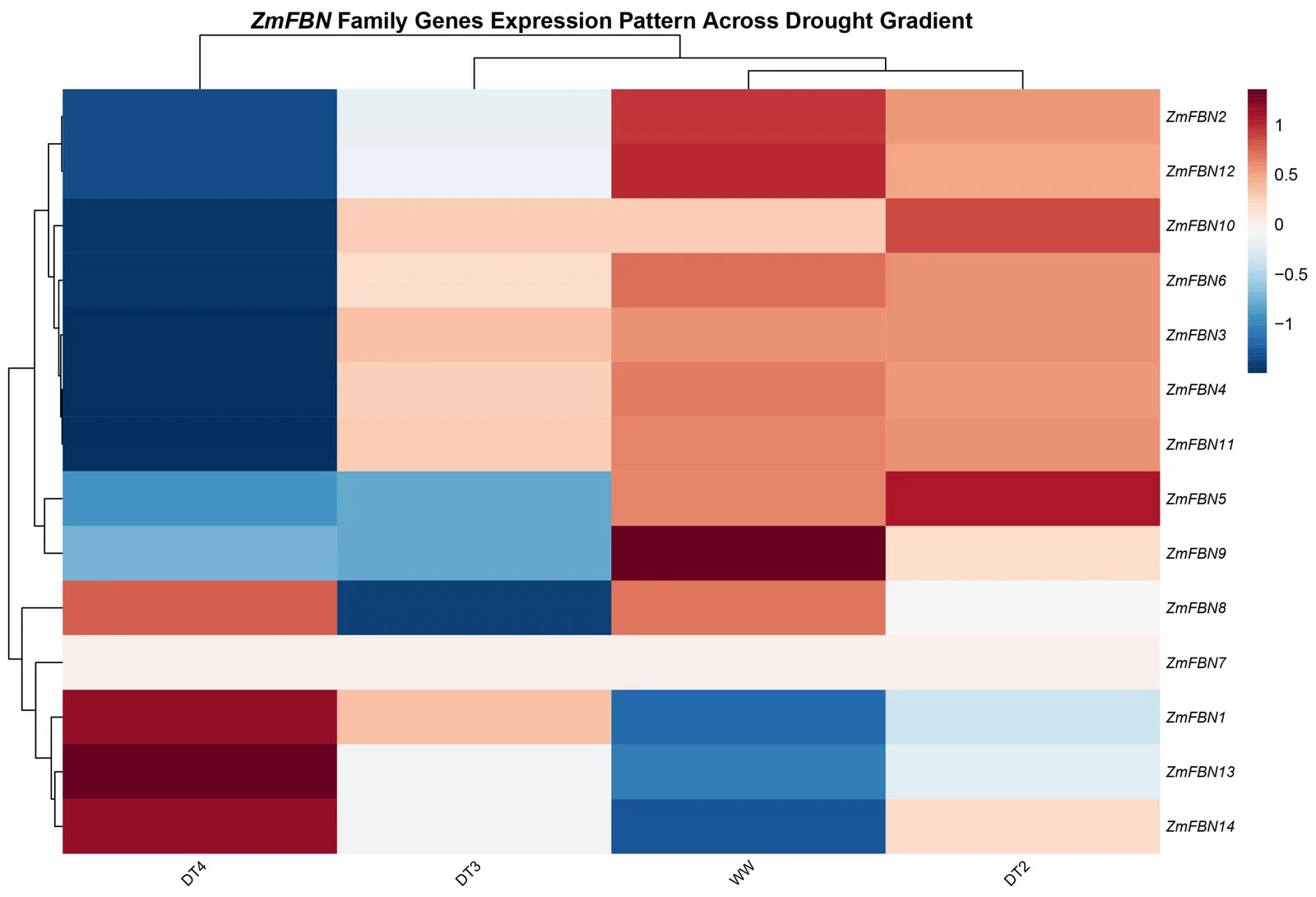
Expression profiles of *ZmFBN* genes under four drought treatments. Transcriptome data were obtained from a published maize drought study [39]. The color scale indicates the expression level (log_2_(FPKM + 1)), with red and blue representing high and low expression, respectively.

Under drought-gradient conditions, *ZmFBN* genes showed markedly different response patterns (Figure 5). Some genes were strongly induced under more severe drought treatments but remained less expressed under well-watered conditions, indicating that these members may mainly function during drought adaptation under a substantial water deficit. For example, *ZmFBN1*, *ZmFBN13*, and *ZmFBN14* showed relatively higher expression under stronger drought stress and lower expression under well-watered conditions, representing a typical drought-induced pattern. In contrast, genes such as *ZmFBN2*, *ZmFBN10*, *ZmFBN11*, and *ZmFBN12* maintained moderate expression even under non-stress conditions, but they still changed noticeably across drought treatments, suggesting that they may participate in both basal plastid maintenance and stress-responsive regulation. A few genes changed only slightly or responded only to specific drought levels, indicating substantial heterogeneity in family-wide drought responsiveness.

**Figure 6:**
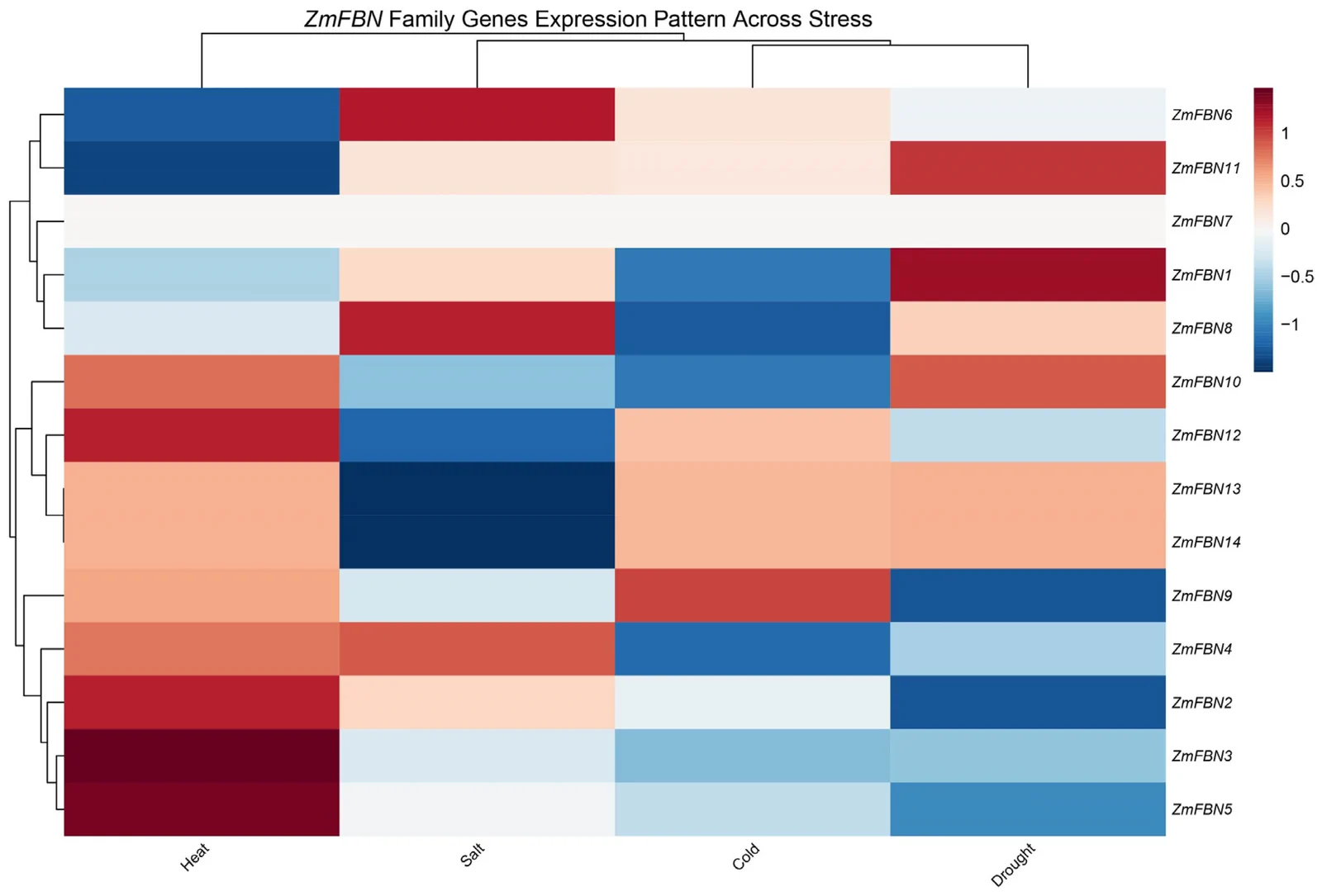
Expression profiles of *ZmFBN* genes under stresses. Transcriptome data from multiple stress treatments were obtained from the MaizeGDB qTeller database. The *x*-axis shows drought (drought stress), salt (salt stress), cold (cold stress) and heat (heat stress), and the *y*-axis lists *ZmFBN* family members. The color scale indicates the expression level (log_2_(FPKM + 1)), with red and blue representing high and low expression, respectively.

Distinct expression divergence was also observed under multiple abiotic stresses (Figure 6). The stress response heatmap shows that *ZmFBN* members responded differently to heat, salt, cold, and drought, indicating that the family does not act as a uniform stress response module. Some genes were preferentially induced by heat and drought, whereas others were more responsive to salt or cold. In particular, *ZmFBN2*, *ZmFBN3*, *ZmFBN5*, and *ZmFBN12* were strongly upregulated under heat stress; *ZmFBN6* and *ZmFBN8* were preferentially induced by salt; and *ZmFBN1* and *ZmFBN9* showed elevated expression under drought and cold, respectively. Some genes displayed complex multi-stress responses: *ZmFBN10* was induced under both heat and drought but suppressed under cold and salt, while *ZmFBN4* was upregulated under heat and salt but downregulated under cold. Notably, only *ZmFBN7* showed minimal expression changes across all four stresses, highlighting the specialized stress-biased roles of most *ZmFBN* genes in abiotic stress signaling pathways. Consistent with its relaxed selective constraint from the Ka/Ks results, the constitutive stable expression of *ZmFBN* across diverse stresses matched its evolutionary feature; meanwhile, positively selected *ZmFBN12* was strongly induced under heat stress, which is consistent with its adaptive evolutionary tendency.

**Figure 7:**
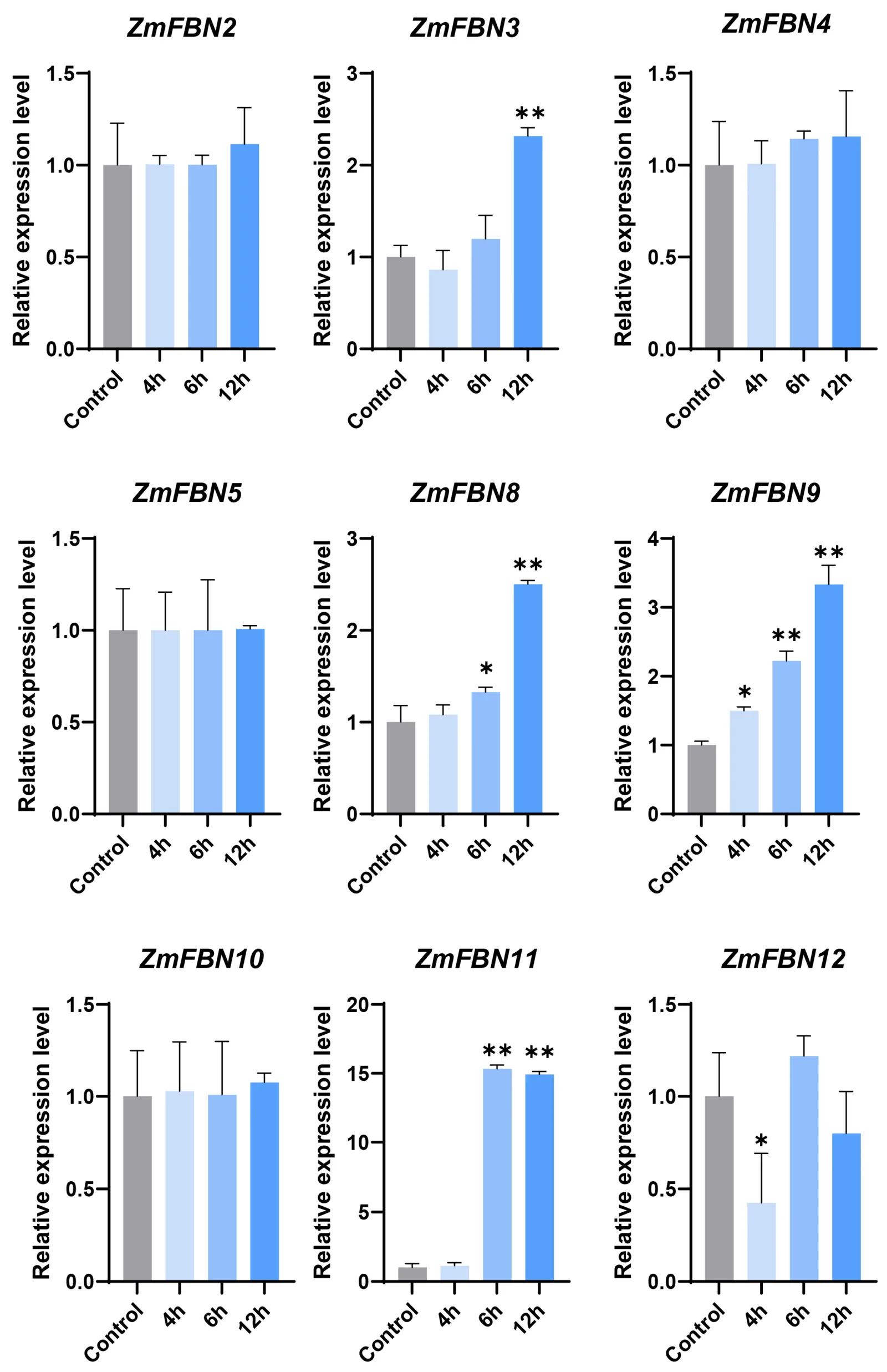
qRT-PCR validation of *ZmFBN* gene expression under salt stress. Three-leaf-stage maize seedlings were irrigated with 200 mM NaCl, and leaf samples (upper third of the third leaf) were collected at 0, 4, 6, and 12 h. Expression levels were normalized to the 0 h control. Three biological replicates (three seedlings per replicate) and three technical replicates were performed. Error bars represent standard deviation (SD) of three biological replicates. Statistical significance was determined by one-way ANOVA with Tukey’s HSD test (* denotes significant (0.01 < *p* < 0.05) ** denotes highly significant (*p* < 0.01), compared with 0 h control).

To validate the transcriptome-based expression profiles, qPCR analysis was performed for selected *ZmFBN* genes under NaCl and PEG treatments. The results were generally consistent with the heatmap-based expression patterns, supporting the reliability of the transcriptomic analysis. Under salt treatment, the selected genes showed distinct and gene-specific expression dynamics during 4–12 h of NaCl exposure (Figure 7). *ZmFBN8*, *ZmFBN9*, and *ZmFBN11* exhibited the most prominent induction, with *ZmFBN8* and *ZmFBN9* showing a progressive increase and reaching their highest levels at 12 h, whereas *ZmFBN11* was strongly upregulated at 6 h and remained highly expressed at 12 h. These results indicate that *ZmFBN8*, *ZmFBN9*, and *ZmFBN11* are highly salt-responsive members and may play important roles in maize salt adaptation. *ZmFBN3* also showed moderate induction, particularly at later time points, suggesting that it is involved in salt response but with lower amplitudes. In contrast, *ZmFBN2*, *ZmFBN4*, *ZmFBN5*, *ZmFBN10*, and *ZmFBN12* exhibited only minor or negligible changes in expression under NaCl treatment, implying that they may contribute to salt responses in a more subtle manner or primarily function in other environmental conditions.

**Figure 8:**
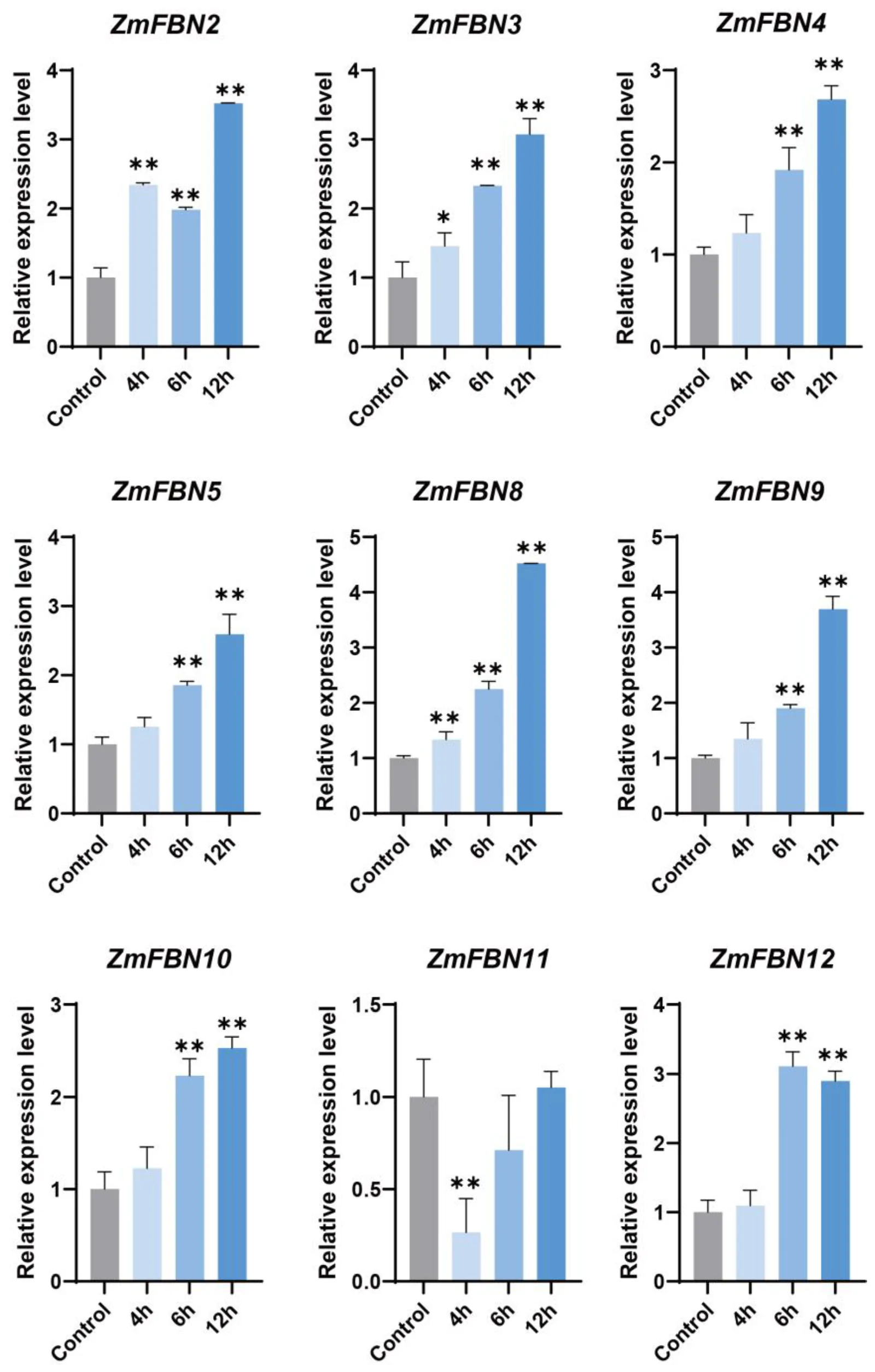
qRT-PCR validation of *ZmFBN* gene expression under PEG-simulated drought stress. Three-leaf-stage maize seedlings were treated with 20% PEG 6000, and leaf samples (upper third of the third leaf) were collected at 0, 4, 6, and 12 h. Expression levels were normalized to the 0 h control. Three biological replicates (three seedlings per replicate) and three technical replicates were performed. Error bars represent standard deviation (SD) of three biological replicates. Statistical significance was determined by one-way ANOVA with Tukey’s HSD test (* denotes significant (0.01 < *p* < 0.05) ** denotes highly significant (*p* < 0.01), compared with 0 h control).

Under PEG-simulated drought treatment, all the tested *ZmFBN* genes showed distinct time-dependent expression changes (Figure 8), with most exhibiting significant upregulation across the treatment period. Most genes, including *ZmFBN2*, *ZmFBN3*, *ZmFBN4*, *ZmFBN5*, *ZmFBN8*, *ZmFBN9*, and *ZmFBN10*, were upregulated to varying degrees, peaking at 12 h; among these, *ZmFBN8* showed the strongest drought response, rising sharply to ~4.5-fold the control level by 12 h, while *ZmFBN2*, *ZmFBN3*, and *ZmFBN9* were significantly induced as early as 4–6 h and remained elevated through 12 h. *ZmFBN4*, *ZmFBN5*, and *ZmFBN10* displayed a delayed response with no significant change at 4 h but marked upregulation from 6 h onward. *ZmFBN12* showed a unique profile, remaining stable at 4 h, peaking at over 3-fold the control level at 6 h, and staying significantly elevated despite a slight decline at 12 h. In contrast, *ZmFBN11* was significantly downregulated at 4 h before returning to near control levels by 6–12 h, suggesting it may play only a transient context-specific role in the early drought response rather than contributing to sustained drought adaptation.

Taken together, the tissue expression analysis, stress expression profiles, and qPCR results consistently demonstrate that *ZmFBN* genes are transcriptionally diversified in maize. On the one hand, many members are highly expressed in leaves and tissues across different developmental stages, including vegetative tissues (root, stem, leaf) and reproductive tissues (tassel, ear, silk, endosperm, embryo), supporting roles in organ development. On the other hand, different genes show distinct response amplitudes and temporal patterns under drought and salt treatments, indicating functional specialization in stress adaptation. In particular, *ZmFBN8* and *ZmFBN9* show sustained induction under both drought and salt conditions and thus represent strong candidates for shared drought/salt responses. In contrast, *ZmFBN2* shows stronger induction under heat and PEG-simulated drought than under salt stress, while *ZmFBN5* displays the most prominent induction specifically under drought. *ZmFBN4* and *ZmFBN10* exhibited inconsistent expression patterns between the transcriptome and qPCR results under salt treatment, with negligible expression changes observed in the qPCR validation.

### 3.4. GWAS-Based Identification of Drought-Related Candidate ZmFBN Genes

To further assess the relationship between the *ZmFBN* family and drought-related traits at the natural population level, GWAS signals around *ZmFBN* loci were examined. Significant association peaks were detected near both *ZmFBN1* and *ZmFBN4*, corresponding to chr1.S_101267108 and chr2.S_11367217, respectively. These results indicate that some *ZmFBN* members are not only stress-responsive at the transcriptional level but may also contribute to phenotypic variation in drought tolerance through natural genetic variation.

**Figure 9:**
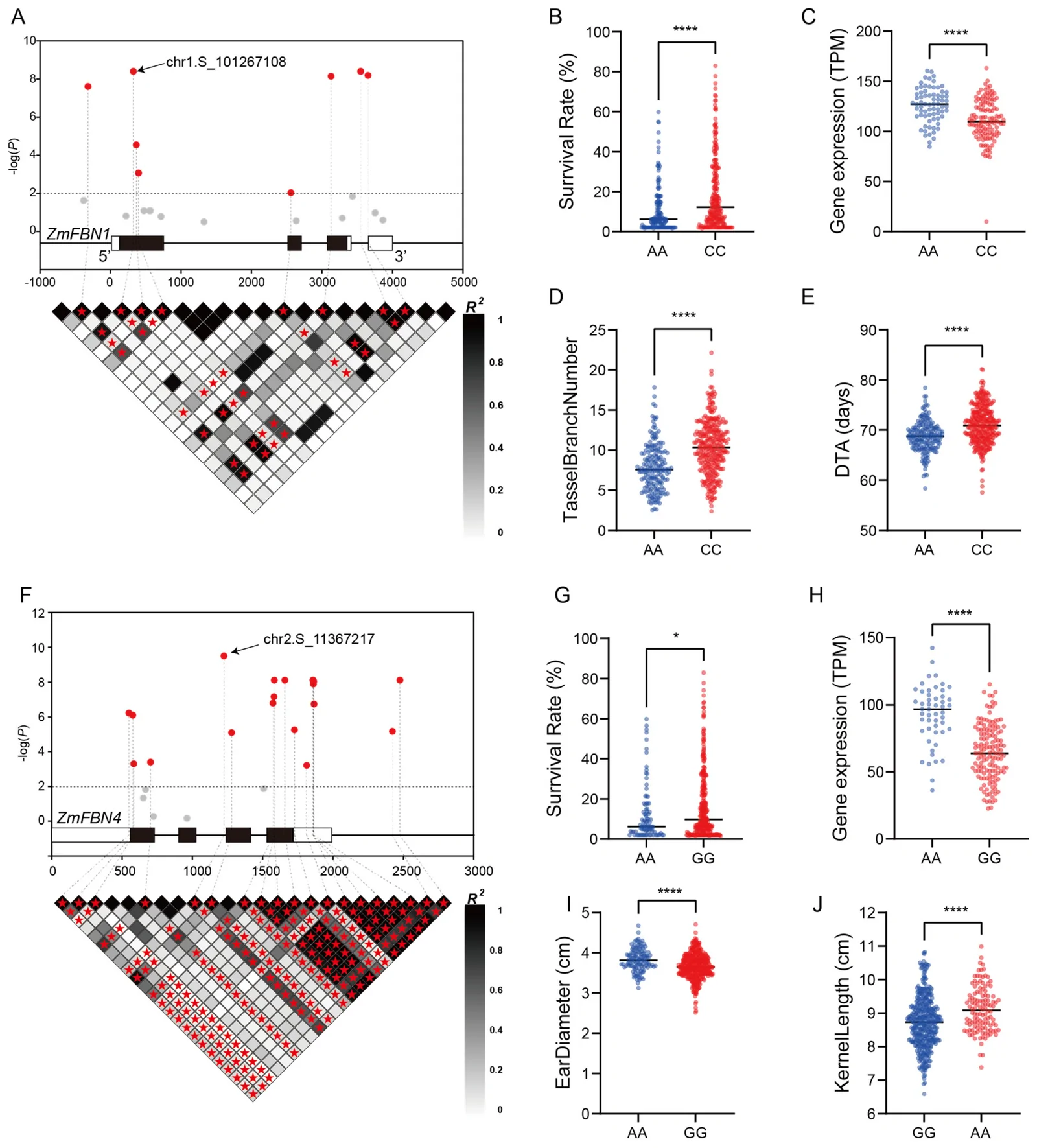
Associations of natural *ZmFBN1* and *ZmFBN4* variants with drought tolerance and agronomic traits in maize. (**A**,**F**) Local association signals and linkage disequilibrium (LD) patterns surrounding the *ZmFBN1* (**A**) and *ZmFBN4* (**F**) loci. The upper panels show –log_10_(*p*) values of SNPs within each gene and its flanking regions; red and gray circles represent SNPs above and below the significance threshold, respectively, and the horizontal dotted line indicates the significance threshold, with the lead SNPs marked by vertical dotted lines, and the lower panels depict the corresponding LD heatmaps (*r*^2^). In the LD heatmaps, the white-to-black gradient represents pairwise *r*^2^ values from 0 to 1, and red stars mark the significant SNPs. (**B**–**E**, **G**–**J**) Phenotypic effects of the lead SNPs in *ZmFBN1* and *ZmFBN4* on drought tolerance and key agronomic traits in maize. Box plots show the distribution of trait values for each allelic class; asterisks indicate significant differences between genotypes (* *p* < 0.05, and **** *p* < 0.0001, Student’s *t*-test).

In the *ZmFBN1* region, local association analysis showed a clear peak around chr1.S_101267108, and the surrounding LD heatmap revealed a relatively strong linkage block (Figure 9A), which suggests that the associated region contains a stable genetic structure that may harbor functional variation linked to drought-related traits. Allelic effect analysis further showed that different genotypes at this locus were significantly associated with multiple traits. Specifically, the AA and CC genotypes differed significantly in the survival rate (Figure 9B), gene expression (Figure 9C), tassel branch number (Figure 9D), and days to anthesis (DTA) (Figure 9E). According to the box plots, the CC genotype tended to show a higher survival rate and tassel branch number, whereas the AA genotype exhibited relatively higher expression levels. These findings suggest that variation near *ZmFBN1* may influence both drought tolerance and developmental traits through effects on gene expression.

A similar pattern was observed in the *ZmFBN4* region. A significant association signal was detected at chr2.S_11367217, and the neighboring region also showed a clear LD structure (Figure 9F). Further analysis indicated that the AA and GG genotypes differed significantly in the survival rate (Figure 9G), gene expression (Figure 9H), ear diameter (Figure 9I), and kernel length (Figure 9J). The AA genotype showed a higher survival rate and expression level than the GG genotype, while the kernel length also differed significantly between the two allelic groups. These results suggest that *ZmFBN4* may be an important candidate gene linking drought adaptation with agronomic trait variation.

Importantly, both *ZmFBN1* and *ZmFBN4* were supported by multiple lines of evidence, including significant GWAS associations, drought-responsive expression patterns, and genotype-dependent differences in gene expression and phenotype. The convergence of the association, expression, and phenotypic data strengthens the reliability of these two genes as drought-related candidates. Therefore, *ZmFBN1* and *ZmFBN4* represent the most promising drought-associated members identified in this study and warrant further functional validation.

### 3.5. Putative Transcriptional Regulatory Network of ZmFBN Genes

**Figure 10:**
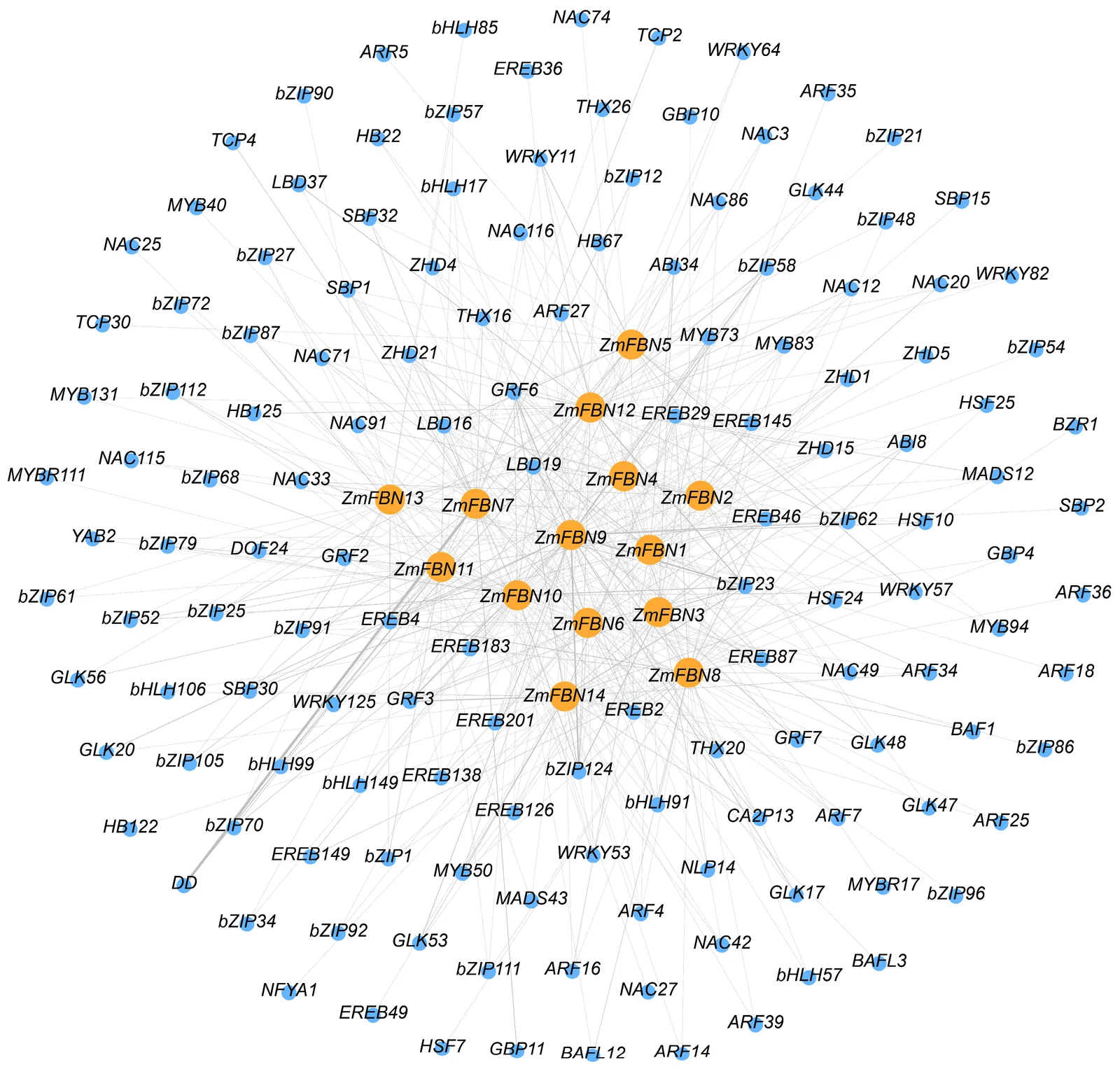
Visualization of the interaction network between upstream transcription factors and target *ZmFBN* family genes. Orange nodes represent *ZmFBN* target genes, and blue nodes represent predicted upstream transcription factors. The lines between nodes represent the direct or indirect regulatory interaction relationships between transcription factors and target genes.

To investigate the upstream regulatory landscape of the *ZmFBN* family, a putative transcription factor regulatory network was constructed (Figure 10). The network showed that the 14 *ZmFBN* genes were connected with a large number of transcription factors, indicating that the family is likely embedded in a complex multilayered regulatory system rather than being controlled by a single signaling pathway. The connectivity of individual *ZmFBN* genes varied considerably, suggesting that different members may occupy different regulatory positions in the network.

The predicted upstream regulators belonged mainly to the *ERF*, *WRKY*, *NAC*, *MYB*, *bZIP*, *ARF*, *DOF*, *SBP*, *TCP*, *GLK*, and *B3* families. Many of these transcription factor families are well known for their roles in abiotic stress signaling, developmental regulation, and hormone-mediated responses, implying that *ZmFBN* genes may integrate developmental and stress-related cues. In particular, the dense connections with *ERF*, *WRKY*, *NAC*, and *bZIP* factors strongly support the involvement of *ZmFBN* genes in abiotic stress response pathways.

The network predictions were also supported by promoter cis-element analysis. Many *ZmFBN* promoters contained MYB-binding sites as well as stress-, hormone-, and defense-related cis-elements, which is highly consistent with the TF families identified in the network. Therefore, the combined promoter and network analyses suggest that *ZmFBN* genes are likely co-regulated by multiple stress- and development-related pathways.

## 4. Discussion

### 4.1. The ZmFBN Family Is Evolutionarily Conserved in Maize but Has Undergone Structural and Functional Diversification

A total of 14 *ZmFBN* genes were identified in the maize genome, indicating that the *ZmFBN* family represents a relatively small but structurally conserved gene family. Phylogenetic analysis showed that *ZmFBN* proteins clustered together with homologs from Arabidopsis, sorghum, and *Setaria italica*, rather than forming a fully independent maize-specific branch, which is consistent with the view that the FBN family originated early and retained core functions during plant evolution. This evolutionary conservation suggests that *ZmFBN* genes likely maintain fundamental roles associated with plastid stability, photosynthetic protection, and stress adaptation in maize.

At the same time, the *ZmFBN* family is clearly not uniform functionally. In this study, substantial differences were observed among *ZmFBN* genes in exon–intron organization, motif composition, chromosomal distribution, and promoter cis-elements. Collinearity among some members suggests that segmental duplication may have contributed to the retention and diversification of parts of the family. Moreover, genes in the same phylogenetic subgroup generally share similar structural features, whereas genes in different branches differ substantially in motif organization and regulatory elements. These findings support a model in which the *ZmFBN* family retained a conserved structural core, while gradually acquiring distinct regulatory and functional characteristics.

Previous studies have shown that FBN proteins are broadly involved in plastoglobule formation, carotenoid accumulation, lipid metabolism, photosystem protection, and oxidative stress buffering [9,45,46]. These processes are directly relevant not only to plastid maintenance but also to crop performance under drought, salinity, and other environmental stresses. Therefore, from an agronomic perspective, the significance of studying the *ZmFBN* family extends beyond family annotation and provides a useful framework for identifying candidate genes associated with maize stress adaptation and stable production. Consistent with sequence-level evidence, *ZmFBN7* with relaxed selection displayed invariant expression under multiple abiotic treatments, while positively selected *ZmFBN12* was strongly upregulated upon heat stress, supporting their functional divergence at the transcriptional level.

### 4.2. Expression Divergence of ZmFBN Genes Suggests Coordinated Roles in Chloroplast Maintenance, Organ Development, and Stress Adaptation

Tissue expression profiling showed that most *ZmFBN* genes were highly expressed in leaves, whereas some members also showed relatively high expression in tassel, embryo, pericarp, silk, and pre-pollination cob-related tissues. These findings suggest that the *ZmFBN* family is associated not only with plastid maintenance in vegetative tissues but also with reproductive development and kernel-related biological processes. Because leaves represent the principal site of chloroplast abundance and photosynthetic activity, the high expression of many *ZmFBN* genes in leaves strongly supports their involvement in chloroplast development, photosystem stability, and photoprotection.

The expression of some *ZmFBN* genes in development-related tissues further suggests broader agronomic relevance. For example, relatively high expression in meiotic tassel, SAM and young SAM, embryo, and pre-pollination cob, together with the presence of seed-specific, endosperm-related, and hormone-responsive cis-elements, implies that some *ZmFBN* genes may contribute to tassel differentiation, reproductive development, and kernel formation. This is particularly relevant for crop studies, because stress-responsive genes that also influence reproductive development may have direct consequences for yield formation and stress–growth trade-offs.

Under drought-gradient and multiple abiotic stress treatments, the *ZmFBN* family displayed substantial expression divergence. Some members were continuously induced under severe drought or under both drought and salt conditions, whereas others responded more strongly to heat or cold. This pattern indicates that the *ZmFBN* family does not operate as a single response unit but rather as a set of genes with partially overlapping yet distinct roles in different stress pathways. Such diversity is consistent with the current understanding that maize responses to drought, salinity, heat, and cold involve both stress-specific and partially shared regulatory modules.

The observation that some *ZmFBN* genes showed similar expression patterns under both drought and salt stress is particularly noteworthy. These genes may participate in shared adaptive processes such as osmotic adjustment, ROS detoxification, membrane stabilization, and ABA-related signaling. In agricultural production systems, drought and salinity often occur simultaneously or sequentially, especially under water-limited or secondary salinization conditions. Therefore, *ZmFBN* genes that respond to both stresses may be especially valuable as broad-spectrum targets for crop stress improvement.

### 4.3. qPCR Validation and Regulatory Network Analysis Reveal Shared and Stress-Specific Response Characteristics of ZmFBN Genes

It should be noted that only a subset of *ZmFBN* family members was subjected to qRT-PCR validation. *ZmFBN1*, despite being a promising GWAS candidate, was excluded because specific qRT-PCR primers could not be reliably amplified in repeated attempts. Other unmeasured genes either failed primer amplification or showed no significant expression variation under drought and salt stress in the RNA-seq data. The qPCR results further confirmed that *ZmFBN* genes exhibit distinct expression patterns under drought and salt stress. In particular, *ZmFBN8* and *ZmFBN9* showed strong induction under NaCl treatment, whereas *ZmFBN5* displayed the most pronounced and sustained induction under PEG treatment. These differences indicate that the response strength and temporal dynamics of *ZmFBN* genes vary substantially between drought and salt conditions. Rather than being governed by a single dominant family member, the *ZmFBN* family appears to function as a coordinated module composed of genes with different stress response preferences.

From an application perspective, this pattern is highly informative. For salt tolerance improvement, *ZmFBN8*, *ZmFBN9* and *ZmFBN11* may represent particularly promising candidates, whereas *ZmFBN5* may be more relevant for drought-related improvement. Meanwhile, *ZmFBN8* and *ZmFBN9*, which are consistently induced under both drought and salt conditions, may represent stronger candidates for broad-spectrum stress adaptation. This distinction suggests that candidate prioritization should not rely solely on the absolute induction level but should also consider whether a gene shows shared responsiveness or stress-specific specialization.

The regulatory network analysis further supports this interpretation. Multiple *ZmFBN* genes were predicted to be connected with transcription factor families such as *ERF*, *WRKY*, *NAC*, *MYB*, *bZIP*, *ARF*, and *TCP*, most of which are well known for their roles in abiotic stress response, hormone signaling, and developmental control. This suggests that the *ZmFBN* family may lie at the intersection of developmental regulation and environmental adaptation rather than functioning solely as downstream stress markers.

This view is reinforced by the promoter analysis, which revealed widespread enrichment of ABA-responsive, MeJA-responsive, MYB-binding, defense-related, and stress-related cis-elements, indicating that *ZmFBN* genes are likely integrated into a complex regulatory framework involving multiple hormones, transcription factors, and environmental signals.

These predicted TF families are well-characterized regulators of abiotic stress: ERF/AP2 (ABA-independent drought signaling via the DREB subfamily), WRKY (stress-responsive transcriptional activation), NAC (drought-induced stomatal closure, e.g., *ZmSNAC1*), and MYB/bZIP (ABA-dependent signaling via ABRE binding). Notably, these ChIP-seq-predicted TF families are concordant with the cis-elements independently identified in *ZmFBN* promoters (ABRE, MBS, W-box, TC-rich repeats), providing cross-validation between network inference [44] and promoter motif analysis. Together, these results indicate that *ZmFBN* genes are likely activated through ABA- and MeJA-dependent transcriptional cascades, with gene-specific TF wiring explaining the differential responsiveness of individual *ZmFBN* members.

Notably, *ZmActin*-1 (Zm00001eb348450) was used as the sole reference gene for RT-qPCR quantification in this study under moderate short-term salt and drought treatment. Although consistent expression trends were observed across biological replicates, the previous literature has reported unstable expression of actin under certain extreme stress environments, which constitutes a methodological limitation of the present research [47]. We recommend that future related studies screen and validate multiple candidate reference genes jointly via algorithms including GeNorm and NormFinder to eliminate quantification bias caused by single internal reference and improve the reliability of qPCR data. Taken together, integrated evidence from qPCR quantification, cis-element identification and regulatory network prediction demonstrates that different *ZmFBN* paralogs have evolved divergent response modes, including drought-specific, salt-specific and broad-spectrum stress-responsive patterns, which provides targeted gene resources for maize stress-resistance molecular breeding.

### 4.4. GWAS Results Identify ZmFBN1 and ZmFBN4 as Key Candidates with Both Molecular and Agronomic Relevance

For a crop-focused study, expression responsiveness alone is often insufficient to establish agronomic value. Integrating GWAS with expression analysis provides a stronger basis for linking candidate genes to field-relevant traits. In this study, significant GWAS signals were detected near *ZmFBN1* and *ZmFBN4*, and different allelic groups at these loci were significantly associated with the survival rate, gene expression, and several agronomically relevant traits. It should be emphasized that these results only demonstrate statistical associations at the population level and cannot directly prove that the variation in *ZmFBN* genes is the causal functional variation for the observed phenotypic differences. Further functional validation via gene editing, overexpression, or mutant analysis is required to confirm the causal relationship.

Among them, *ZmFBN4* appears to be one of the strongest candidate genes identified in this study. First, it is located within a significant GWAS region and is associated with the survival rate, gene expression, ear diameter, and kernel length. Second, it showed stable induction under drought treatment and was supported by qPCR validation, while its salt responsiveness was not confirmed in the qPCR assays. This combination of association, expression, and phenotypic evidence suggests that *ZmFBN4* may simultaneously contribute to stress adaptation and agronomic trait regulation, making it a high-priority target for future functional validation.

The candidacy of *ZmFBN1* and *ZmFBN4* is supported by multiple converging lines of evidence: (i) significant GWAS signals at the natural population level; (ii) stable LD haplotype blocks surrounding both loci (Figure 9A,F); (iii) genotype-dependent differences in survival rate under drought stress, with ZmFBN4 additionally associated with ear diameter and kernel length (Figure 9B–E,G–J); and (iv) drought-responsive expression in public RNA-seq data (Figure 5). For *ZmFBN4*, drought responsiveness was independently confirmed by qRT-PCR under PEG treatment (Figure 8); for *ZmFBN1*, qRT-PCR data are not yet available in the present study. The functional relevance of both candidates is further supported by cross-species evidence: the PAP-fibrillin domain they share mediates plastoglobule localization in Arabidopsis and tomato, where homologous FBN proteins function in carotenoid sequestration, membrane stabilization, and oxidative stress buffering.

*ZmFBN1* showed clear signals in GWAS, local LD structure, and allelic effect analyses. This observation highlights an important point in crop gene discovery: the most biologically or agronomically relevant gene is not necessarily the one with the highest transcriptional induction under controlled stress treatment. Some genes may exert stronger effects through constitutive expression, developmental regulation, or naturally occurring allelic variation that influences field adaptation. Therefore, *ZmFBN4* should be regarded as the top-priority candidate in this study, whereas *ZmFBN1* should be considered an important candidate with strong population genetic support.

Based on these converging lines of evidence, we propose that stress-induced *ZmFBN* expression enhances plastid membrane stabilization and oxidative stress buffering, thereby protecting photosynthetic capacity under drought and salt stress. This working model is presented as a testable hypothesis that requires direct functional validation.

### 4.5. Significance, Limitations, and Implications for Maize Stress Resilience Improvement

This study systematically investigated the *ZmFBN* family at multiple levels, including family identification, structural characterization, tissue-specific expression, stress-responsive expression, qPCR validation, GWAS association, and regulatory network prediction. Compared with studies that focus only on family annotation or expression description, the integration of qPCR validation and GWAS in this work provides stronger support for identifying candidate genes with potential relevance for maize stress improvement.

Nevertheless, several limitations should be acknowledged. First, functional inference in this study is mainly based on bioinformatic analyses, expression responses, and association signals; experimental verification of subcellular localization was not performed for all family members in this genome-wide survey, and direct genetic evidence from overexpression, knockout, genome editing, or mutant analysis is lacking. Second, FBN proteins are likely to influence stress adaptation through plastoglobule ultrastructure, lipid remodeling, carotenoid accumulation, and ROS homeostasis; however, these physiological and cellular mechanisms were not directly examined here. Third, both the regulatory network and cis-element analyses represent predicted relationships that require validation through experiments such as Y1H, dual-luciferase assays, EMSA, or ChIP-qPCR.

Therefore, future studies should focus on three aspects. First, high-priority candidates such as *ZmFBN4* and *ZmFBN1* and the strongly responsive genes *ZmFBN5*, *ZmFBN8*, *ZmFBN9*, and *ZmFBN12* should be functionally validated to determine their actual contributions to drought and salt tolerance. Second, these genes should be examined in combination with the chloroplast ultrastructure, ROS status, osmotic adjustment, and photosynthetic traits to clarify their physiological roles in stress adaptation. Third, superior haplotypes and allelic variants should be further explored in natural populations, and breeder-friendly markers should be developed to facilitate the transition from candidate gene identification to maize stress resilience improvement.

## Data Availability

All data generated or analyzed during this study are included in this published article. Additional datasets supporting the findings of this study are available from the corresponding authors upon reasonable request.

## Supplementary Materials

The following supporting information can be downloaded at: https://www.mdpi.com/article/10.3390/cimb48080819/s1.
